# Substrate binding and catalytic mechanism of UDP-α-D-galactofuranose: β-galactofuranoside β-(1→5)-galactofuranosyltransferase GfsA

**DOI:** 10.1093/pnasnexus/pgae482

**Published:** 2024-10-25

**Authors:** Takuji Oka, Ayana Okuno, Daisuke Hira, Takamasa Teramoto, Yuria Chihara, Rio Hirata, Chihiro Kadooka, Yoshimitsu Kakuta

**Affiliations:** Department of Biotechnology and Life Sciences, Faculty of Biotechnology and Life Sciences, Sojo University, 4-22-1 Ikeda, Nishi-ku, Kumamoto 860-0082, Japan; Laboratory of Biophysical Chemistry, Department of Bioscience and Biotechnology, Faculty of Agriculture, Kyushu University, 744 Motooka, Nishi-ku, Fukuoka 819-0395, Japan; Department of Biotechnology and Life Sciences, Faculty of Biotechnology and Life Sciences, Sojo University, 4-22-1 Ikeda, Nishi-ku, Kumamoto 860-0082, Japan; Laboratory of Biophysical Chemistry, Department of Bioscience and Biotechnology, Faculty of Agriculture, Kyushu University, 744 Motooka, Nishi-ku, Fukuoka 819-0395, Japan; Department of Biotechnology and Life Sciences, Faculty of Biotechnology and Life Sciences, Sojo University, 4-22-1 Ikeda, Nishi-ku, Kumamoto 860-0082, Japan; Laboratory of Biophysical Chemistry, Department of Bioscience and Biotechnology, Faculty of Agriculture, Kyushu University, 744 Motooka, Nishi-ku, Fukuoka 819-0395, Japan; Department of Biotechnology and Life Sciences, Faculty of Biotechnology and Life Sciences, Sojo University, 4-22-1 Ikeda, Nishi-ku, Kumamoto 860-0082, Japan; Laboratory of Biophysical Chemistry, Department of Bioscience and Biotechnology, Faculty of Agriculture, Kyushu University, 744 Motooka, Nishi-ku, Fukuoka 819-0395, Japan

**Keywords:** galactofuranosyltransferase, galactofuranose, pathogenic fungi, catalytic mechanism, *Aspergillus fumigatus*

## Abstract

UDP-α-D-galactofuranose (UDP-Galf): β-galactofuranoside β-(1→5)-galactofuranosyltransferase, known as GfsA, is essential in synthesizing β-(1→5)-galactofuranosyl oligosaccharides that are incorporated into the cell wall of pathogenic fungi. This study analyzed the structure and function of GfsA from *Aspergillus fumigatus*. To provide crucial insights into the catalytic mechanism and substrate recognition, the complex structure was elucidated with manganese (Mn^2+^), a donor substrate product (UDP), and an acceptor sugar molecule (β-galactofuranose). In addition to the typical GT-A fold domain, GfsA has a unique domain formed by the N and C termini. The former interacts with the GT-A of another GfsA, forming a dimer. The active center that contains Mn^2+^, UDP, and galactofuranose forms a groove structure that is highly conserved in the GfsA of Pezizomycotina fungi. Enzymatic assays using site-directed mutants were conducted to determine the roles of specific active-site residues in the enzymatic activity of GfsA. The predicted enzyme–substrate complex model containing UDP-Galf characterized a specific β-galactofuranosyltransfer mechanism to the 5ʹ-OH of β-galactofuranose. Overall, the structure of GfsA in pathogenic fungi provides insights into the complex glycan biosynthetic processes of fungal pathogenesis and may inform the development of novel antifungal therapies.

**Significance Statement**

Glycosyltransferases synthesize complex glycans and play crucial roles in cellular functions. Filamentous fungi synthesize sugar chains containing galactofuranose, which are incorporated into the cell wall. GfsA is an enzyme that transfers β-galactofuranose from UDP-α-D-galactofuranose to the 5ʹ-OH of β-galactofuranose. Here, we describe the crystal structure of GfsA from GT31 complexed with manganese ion, UDP, and galactofuranose, revealing a dimeric structure with a unique domain in addition to the typical GT-A fold. This insight enhances our understanding of fungal galactofuranosylation and provides a foundation for further studies on glycan biosynthesis pathways with potential implications for future antifungal research.

## Introduction

Pathogenic fungi can cause significant life-threatening infections in humans and devastating diseases in plants (1). Identifying compounds that are toxic to fungal cells but harmless to human or plant cells is challenging because of their common eukaryotic nature (2). Currently, antifungal treatment options are limited, and the emergence of fungal resistance to azoles, which are key therapeutic agents, is a major public health and agricultural concern (3, 4). Consequently, the development of antifungal therapies for human and crop protection is urgently needed.

Unlike the predominant six-membered ring galactopyranose found in most organisms, galactofuranose (Galf), which forms a five-membered ring, is a monosaccharide absent from humans and plants but incorporated into the cell wall sugar chain of fungi (5–7). Inhibiting the biosynthetic pathway of the Galf-containing sugar chains could assist in developing medicines and agrochemicals without causing adverse effects (5–7). Galf is a key molecule in elucidating the mechanism of pathogenicity of these fungi (8–14). GfsA was the first UDP-α-D-galactofuranose (UDP-Galf): β-galactofuranoside β-(1→5)-galactofuranosyltransferase found in eukaryotes (15–18). A Δ*gfsA* and Δ*gfsC* double mutant in *Aspergillus fumigatus* shows a deficiency in β-(1→5)-galactofuranosyl residues in galactomannan (17). This deficiency reduces the growth rate and conidial formation to increase abnormal hyphal branching structure and cell surface hydrophobicity (17). However, the specific mechanism whereby galactofuranosyltransferases synthesize and incorporate these sugar chains into the cell wall remains unclear. Galactofuranosyltransferases are essential in the synthesis of galactofuranosyl oligosaccharide structures, highlighting their importance in developing therapeutic and agricultural approaches (19).

Only a small number of galactofuranosyltransferases have been characterized in *Mycobacterium tuberculosis*, *Escherichia coli*, *Leishmania donovani*, and *Leishmania major* (20–23). Most research has focused on GlfT2 from *M. tuberculosis*, which is responsible for the synthesis of galactan in the arabinogalactan region of the mycolylarabinogalactan–peptidoglycan complex, an essential component of the cell wall (24). Galactan is a polysaccharide chain composed of Galf units linked by alternating β-(1→5) and β-(1→6) linkages. GlfT2 catalyzes the formation of both β-(1→5) and β-(1→6) linkages (25). The GlfT2 tetrameric structure shows that a single active site can form both types of glycosidic linkages (26), while the enzyme is supposed to operate through a processive mechanism, by consecutively adding multiple Galf units at the same active site, with estimates suggesting the addition of 30–35 Galf units per processive cycle (26). When an acceptor substrate with a β-D-Galf-(1→6)–β-D-Galf-(1→5)–β-D-Galf linkage binds to the active site, the terminal β-(1→6) linkage positions are positioned deeper in the active site and promote the reaction with UDP-Galf at the 5ʹ-OH. Conversely, when an acceptor substrate with a β-D-Galf-(1→5)–β-D-Galf-(1→6)–β-D-Galf linkage binds to the active site, the terminal β-(1→5) linkage positions less deeply in the active site, promoting the reaction with the 6ʹ-OH (27). Unlike GlfT2, GfsA specifically transfers β-Galf to the 5ʹ-OH of the terminal β-galactofuranosyl residue and not to the 6ʹ-OH (16, 17).

In this study, we present the crystal structure of GfsA-Mn^2+^-UDP and Galf, the β-Galf β-(1→5)-galactofuranosyltransferase from *A*. *fumigatus*. The ubiquity of GfsA in a wide range of fungi suggests the possibility of common enzymatic mechanisms or structural features that are essential to inform broader antifungal strategies. By analyzing the crystal structure of the substrate complex and mutants of the active residues, we aim to gain new insights into the enzymatic mechanism and substrate interactions, to advance our knowledge in fungal infection mechanisms, and to clarify the general reaction mechanisms of enzymes involved in sugar biosynthesis and modification.

## Results

### Crystal structure of GfsA

The soluble catalytic domain of GfsA, comprising residues 43–537 with an N-terminal His-tag for purification, was expressed in *E. coli* (16). A gel filtration chromatographic analysis of GfsA purified with Ni-agarose detected a broad single peak with an estimated molecular weight of 120 kDa (Fig. S1A–C), indicating that the 58-kDa GfsA enzyme is predominantly dimeric in solution.

**Fig. 1.**
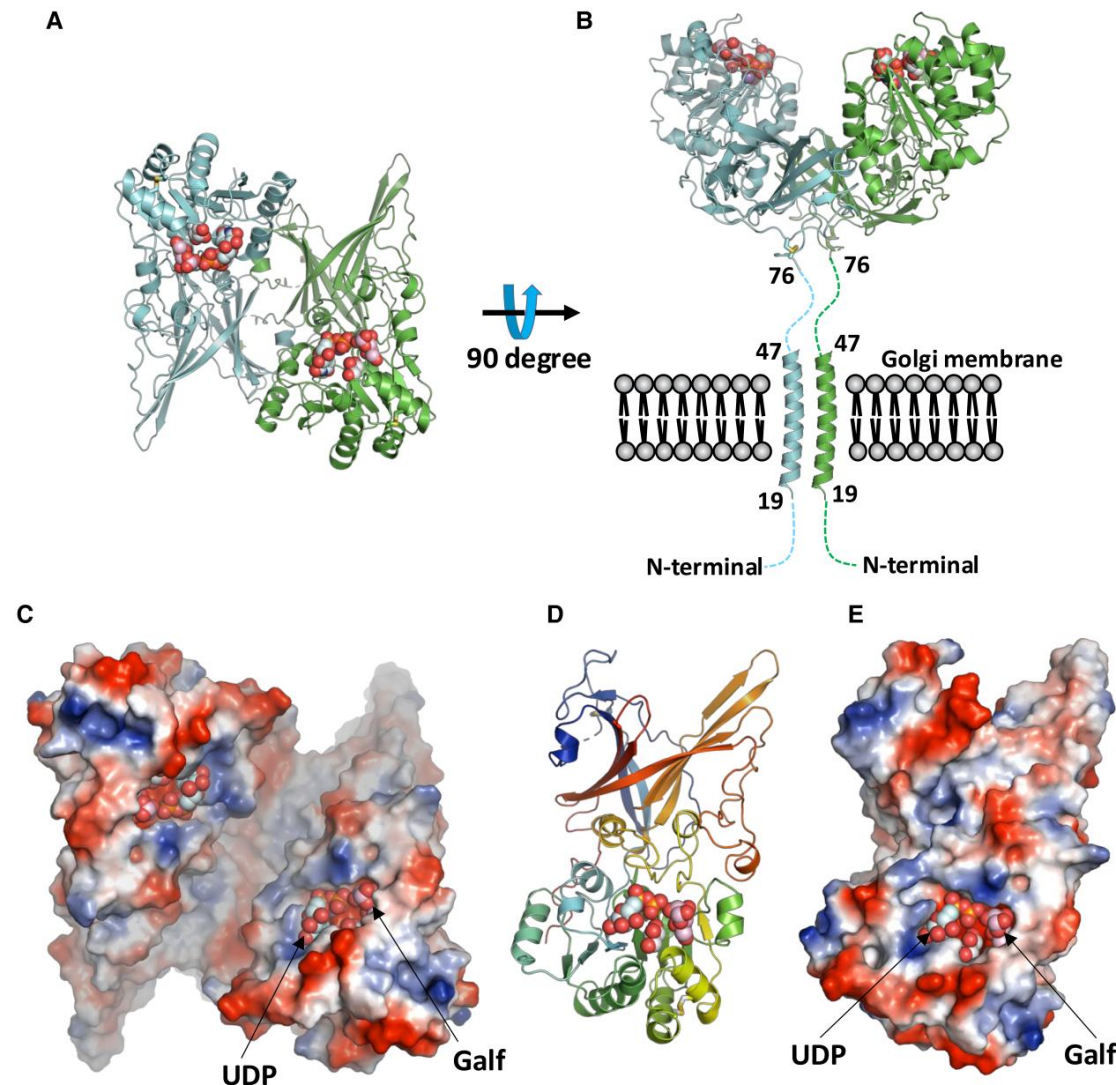
Overall structure of GfsA. A) Dimeric complex of GfsA. Protomers A and B are in green and light blue, respectively. B) The side view of the dimer. N termini of both protomers are sketched as dashed lines. The Golgi membrane is located ipsilateral to the N termini. C) The dimeric complex of GfsA using an electrostatic surface model in the same orientation as (A). D) Ribbon and E) electrostatic surface models of protomer A of GfsA dimer.

The initial structure of GfsA was determined using the single-wavelength anomalous diffraction (SAD) method, using data at 2.99–2.92 Å resolution obtained from an iodine-soaked crystal (Table 1). A diffraction dataset with a resolution of 2.46–2.37 Å was obtained for a crystal of GfsA–Mn^2+^–UDP and Galf complex (Table 1). *Para*-nitrophenyl (pNP)-β-Galf was used in crystallization; however, the pNP group's electron density was unresolved. This may be because the pNP group is not recognized by the enzyme and is highly mobile within the crystal structure. The structure of the GfsA–Mn^2+^–UDP and Galf complex was determined *via* molecular replacement, using the initial structure of GfsA, followed by structure refinement. The complex of GfsA–Mn^2+^–UDP and Galf is dimeric, aligning well with the gel filtration chromatography findings (Figs. 1A and B and S1A–C). Monomers were point-symmetrically attached to each other (Fig. 1C). Amino acids in the dimeric contact surface were detected and identified by PISA. Consurf analysis revealed that amino acids at the dimer contact surface are highly conserved in other fungi-derived GfsA (Fig. S2A–C) (28). Of the amino acids on the dimer contact surface, W92, L93, R133, G13, L183, P184, L187, Y188, G191, Y214, R216, R218, G219, K391, T393, and R401 had a ConSurf score ≥7 (Fig. S2A). Notably, amino acid residues 43–75 of the N-terminal stem region of the crystal structure lacked a discernible electron density, indicating that this N-terminal region does not form stable structure. The two C-terminal amino acid residues also lacked identifiable electron density. The arrangement of both N termini on the same face of the GfsA dimer hinted at a functional orientation, where the dimer could be anchored to the Golgi membrane *via* the N-terminal transmembrane segments (omitted in the expression construct design), positioning the catalytic domain optimally within the Golgi lumen (Fig. 1B). The GfsA monomer comprises two domains (Fig. 1D and E). The region of each domain was predicted using the SWift and Optimized Recognition of protein Domains 2 (SWORD2) tool (29) (Fig. S3A and B). There were a domain with a GT-A fold domain (α-helical domain) that includes the central region (spanning residues 160–360) and a GfsA-specific domain (β-sandwich domain) that includes the N- and C-terminal regions (residues 76–159 and 361–535) (Fig. S3A and B). The GT-A fold domain contains a DXD motif, near where the UDP, Mn^2+^, and Galf are coordinated. The monomer GfsA had two sets of disulfide bonds: C77–C148 and C326–C342.

**Table 1.** Crystallographic summary of GfsA.

Data collection	Iodine derivative	UDP, Mn^2+^, Galf complex
Space group	*P*2_1_2_1_2	*P*2_1_2_1_2
Cell dimensions		
*a*, *b*, c (Å)	82.8, 188.1, 67.3	84.1, 188.6, 71.9
α, β, γ (°)	90.0, 90.0, 90.0	90.0, 90.0, 90.0
Resolution (Å)	47.0–2.92 (2.99–2.92)^a^	47.3–2.37 (2.46–2.37)^a^
Wavelength (Å)	1.3000	1.0000
*R*_sym_	0.299(0.679)^a^	0.087 (0.666)^a^
CC (1/2)	99.9 (96.3)^a^	99.9 (92.7)^a^
*I*/σ*I*	17.8 (3.86)^a^	14.4 (2.55)^a^
Completeness (%)	99.3 (97.7)^a^	99.5 (98.3)^a^
Redundancy	51.1 (14.1)^a^	7.3 (7.3)^a^
**Refinement**		
Resolution (Å)		47.3–2.37 (2.42–2.37)
No. of reflections		47,026 (2,495)^a^
*R*_work_/*R*_free_ (%)		20.59/26.82
No. of atoms		
Protein		7,504
Ligands		142
Waters		318
Average B-factors		
All		46.97
Protein		46.83
Ligands		58.79
Waters		44.30
RMS deviations		
Bond lengths (Å)		0.007
Bond angles (°)		0.492
Ramachandran plot		
Favored (%)		96.92
Allowed (%)		3.08
Outliers (%)		0.00

^a^Values in parentheses are for the highest resolution shell.

### Active-site structure of GfsA

**Fig. 2.**
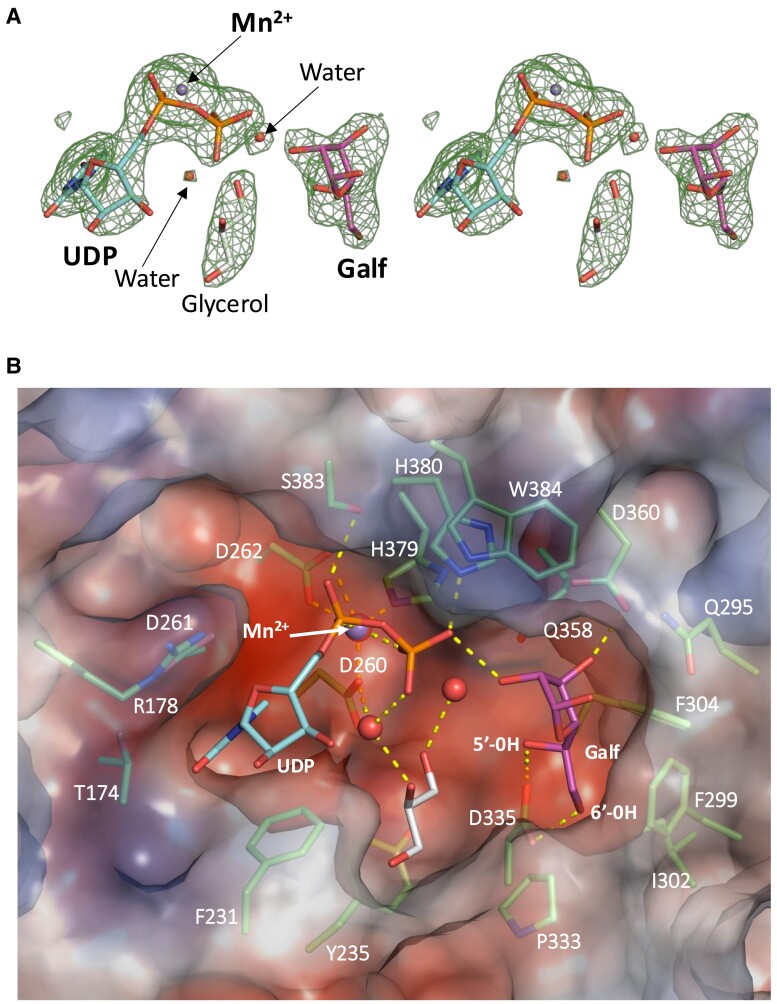
Active site of GfsA. A) Electron density maps of bound UDP–Mn^2+^, Galf, glycerol, and two water molecules. The *F*_o_–*F*_c_ omit map (phenix polder map) contoured at 5σ is superimposed on the molecules. B) Residues, UDP, Gal_f_, and glycerol molecules are shown as stick models and colored in green, light blue, magenta, and white, respectively. Oxygen, nitrogen, and phosphorus are shown in red, blue, and orange, respectively. Mn^2+^ and water molecules are shown in sphere models and colored in purple and red, respectively.

The depiction of the active-site structure of the GfsA complex with UDP, Mn^2+^, and Galf is shown in Fig. 2A and B. While not all GT-A glycosyltransferases require metal ions, bivalent metal ions, such as Mn^2+^, play a crucial role in the reactions mediated by numerous members of GT-A family. Near this region, a recessed space is presumed to be the active site, characterized by a negative charge within the interior (Fig. 2B), while the area surrounding the depression is positively charged (Fig. 2B). The predicted active site of GfsA has a cavity structure comprising Y235, D261, Q295, F299, I302, F304, P333, D335, D360, H379, H380, and W384. Mn^2+^, UDP, Galf, and glycerol were found to bind within the GfsA active site (Fig. 2A). The UDP, a product of the donor substrate, was coordinated with Mn^2+^ through interactions with the DXD motif (D260, D262, and H379) (Fig. 2B). UDP interacted with W384, H380, and S383 regarding the phosphate group, while A173, R178, F231, I259, and D261 interacted with the uracil moiety. L172 and I259 formed hydrogen bonds with water molecules and further interacted with the uracil moiety. The acceptor substrate, Galf, was present within the depression and hydrogen bonded with D335 and D360 (Fig. 2B).

### Mutational analysis

**Fig. 3.**
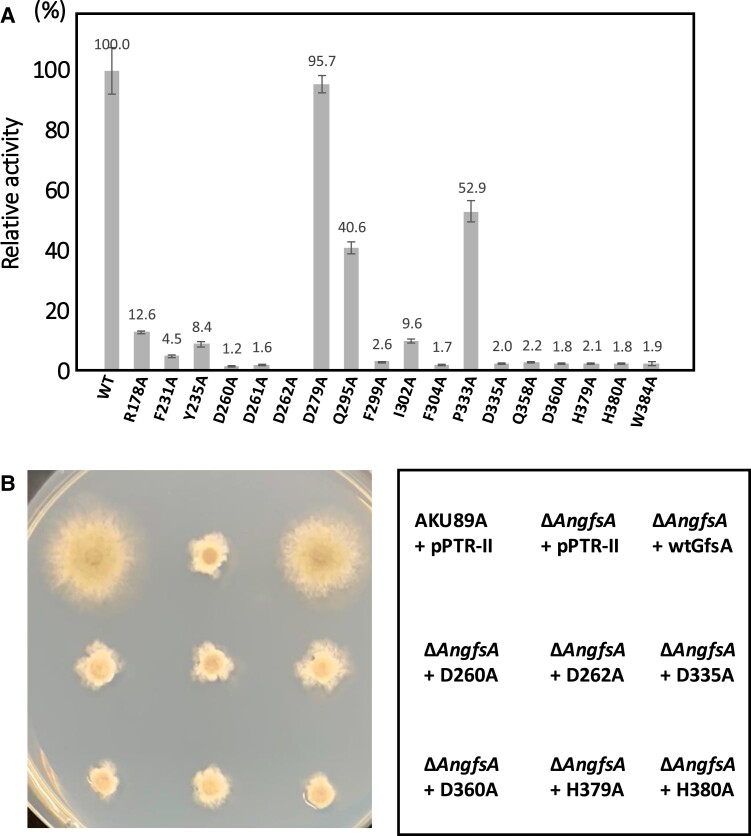
Effect of alanine substitution of amino acids on the activity of *A. fumigatus* GfsA. A) Measurement of GfsA activity using 18 recombinant mutants with alanine substitution at residue positions. WT GfsA and 18 mutants (R178A, F231A, Y235A, D260A, D261A, D262A, D279A, Q295A, F299A, I302A, F304A, P333F, D335A, Q358A, D360A, H379A, H380A, and H384A) were expressed in *E. coli* and purified. The activity of galactofuranosyltransferase was measured; a value of 100% corresponds to the incorporation of 1.8 × 10^−2^ units (nmol/min/μg) of WT GfsA. B) Mycelial growth of *A. nidulans* Δ*gfsA* cells expressing alanine substitutes of *A. fumigatus GfsA*. WT and six mutants (D260A, D262A, D335A, D360A, H379A, and H380A) of GfsA were expressed in *A. nidulans* Δ*gfsA* cells. The empty vector, pPTR-II, was introduced into AKU89A and *A. nidulans* Δ*gfsA* strains.

Based on the crystal structure, we conducted a mutational analysis on residues forming the putative catalytic and Mn^2+^-binding sites (Fig. 3A). To assess the roles of the D260, D262, and H379 residues involved in Mn^2+^ binding, we generated alanine substitution mutants. Additionally, alanine substitution mutants were prepared for Y235, D261, Q295, F299, I302, F304, P333, D335, D360, H380, and W384, which constitute the putative catalytic site, to explore their contributions to the activity. Alanine mutants were also created for R178 and F231 residues that interacted with the uracil moiety and for the D279 residue on the molecular surface opposite the side with the pocket to which the substrate binds. All alanine-substituted recombinants were purified *via* Ni-agarose and subsequently analyzed by SDS–PAGE, followed by Coomassie Brilliant Blue staining. After cell lysis, the lysate was centrifuged at 30,000*×g* for 30 min to obtain the supernatant, ensuring that only soluble proteins were purified using Ni-agarose. SDS–PAGE, in combination with CBB staining, was used to verify that protein sizes were consistent across samples and to assess the protein concentration by comparing the staining intensities. Protein concentrations were normalized to ensure consistency (Fig. S4). Mutations D260A, D262A, and H379A as well as at D261A, F299A, F304A, D335A, Q358A, D360A, H380A, and W384A produced a complete loss of activity (Fig. 3A). Conversely, mutations R178A, F231A, Y235A, and I302A significantly decreased the activity to 12.6, 4.5, 8.4, and 9.6%, respectively, of that of the wild-type GfsA (Fig. 3A). Q295A and P333A mutations had a smaller effect with the activity decreased to 40.6 and 52.9%, respectively (Fig. 3A). The D279A mutation did not affect the activity (Fig. 3A). Next, we conducted in vivo experiments using mutant strains of GfsA (D260A, D262A, D335A, D360A, H379A, and H380A) where the galactofuranosyltransferase activity had been completely lost. These mutants were expressed in the Δ*AngfsA* strain of *Aspergillus nidulans* to assess their functionality in vivo (Fig. 3B). Previous studies have shown that the Δ*AngfsA* strain exhibits suppressed mycelial elongation and reduced conidiophore formation (15). We found that the wild-type GfsA-expressing strain restored mycelial elongation and conidiophore formation to levels similar to that of the parent strain (Fig. 3B), whereas the mutant-expressing strains showed no recovery (Fig. 3B). These results indicate that these specific residues in GfsA are crucial for normal fungal growth.

### Enzyme–substrate complex of GfsA and catalytic mechanism

**Fig. 4.**
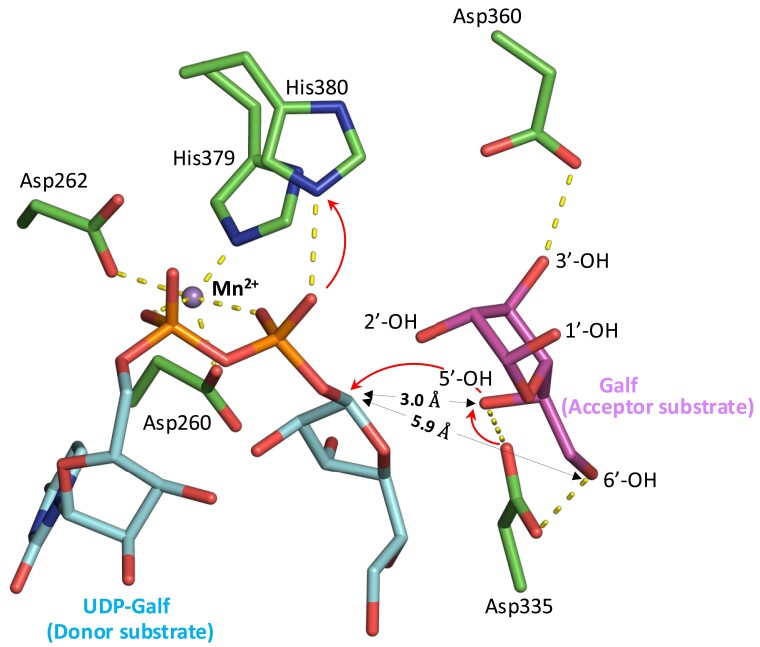
Close-up view of the active site of GfsA. Yellow dotted lines indicate interactions between atoms. The distance between the first position of UDP-Galf and 5ʹ-OH of the accepting substrate Galf is 3.0 Å. The distance between the first position of UDP-Galf and 6ʹ-OH of the accepting substrate Galf is 5.9 Å. Red arrows represent the movement of electrons.

**Fig. 5.**
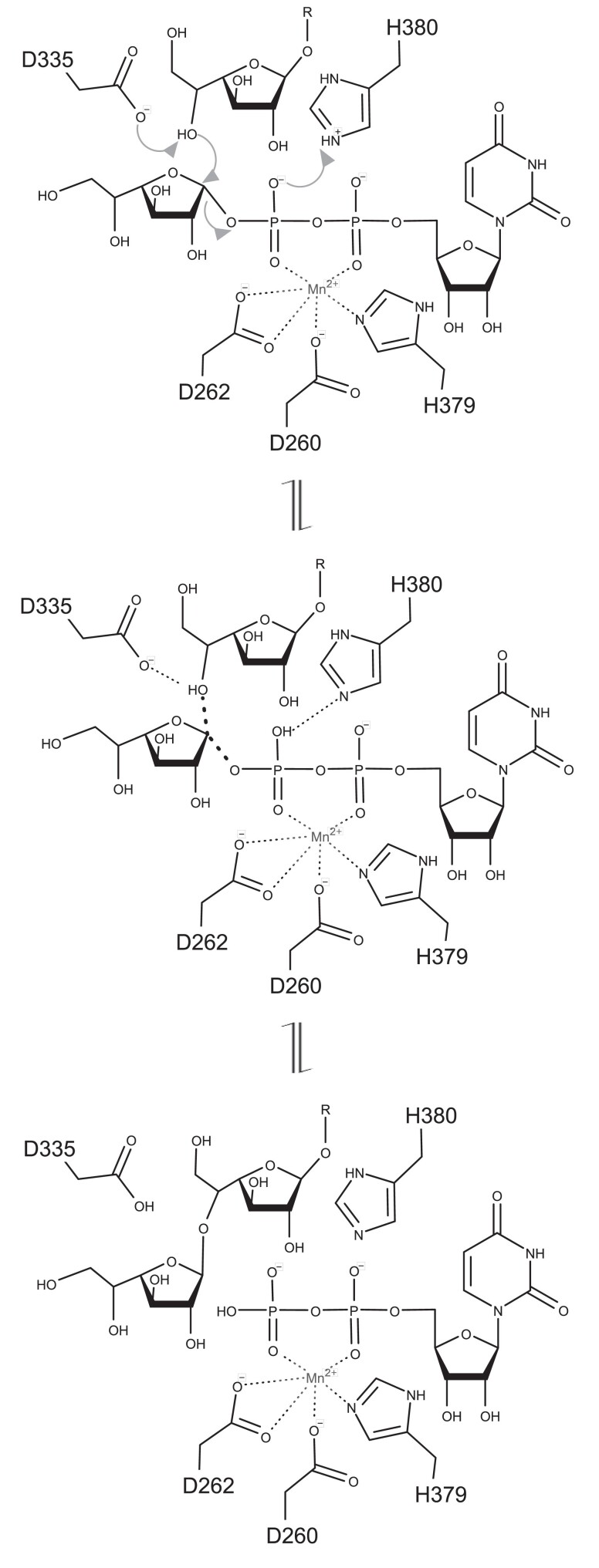
Schematic diagram of the specific transfer mechanism from UDP-galactofuranose to the 5ʹ-OH of the galactofuranose residue by GfsA. Dotted lines indicate the interactions between the atoms. Arrows represent the movement of the electrons.

We then used the crystal structure of GfsA in complex with UDP–Mn^2+^ and the acceptor Galf to model the enzyme–substrate (ES) complex with the donor substrate UDP-Galf (Fig. 4), which was positioned within the active-site cavity (Fig. 4), and proposed a catalytic mechanism for GfsA based on this model (Fig. 5). D360 precisely orients the acceptor substrate by forming a hydrogen bond with the 3ʹ-OH group of the acceptor Galf. D335 assists in deprotonating –OH of the acceptor substrate Galf, which acts as the nucleophile. The specificity of GfsA for the β-(1→5) linkage can be explained by this model (Figs. 4 and 5). D335 interacts with the 5ʹ-OH and 6ʹ-OH groups of the acceptor Galf, and both sites appear capable of being deprotonated. The distance between the 5ʹ-OH of the acceptor substrate Galf and the carbon at position 1 of UDP-Galf is only 3.0 Å, whereas this is 5.9 Å for 6ʹ-OH. Therefore, the 5ʹ-OH, which is closer, is preferentially deprotonated to nucleophilically attack the anomeric carbon of UDP-Galf. Based on the H++ results, the pK(1/2) value for H380 was 8.553, which is substantially higher than the typical histidine pKa of ∼6, indicating that H380 is likely protonated under enzymatic reaction conditions. This observation strongly supports the notion that H380 is protonated. H380 acts as an acid catalyst, donating a proton to the phosphate group of UDP-Galf. GfsA functions as an inverting enzyme, catalyzing the transfer of a Galf residue from UDP-α-Galf to the 5ʹ-OH of a β-galactofuranoside substrate, leading to the formation of a Galf-β-(1→5)-Galf product.

### Comparison with enzymes with similar functions

**Fig. 6.**
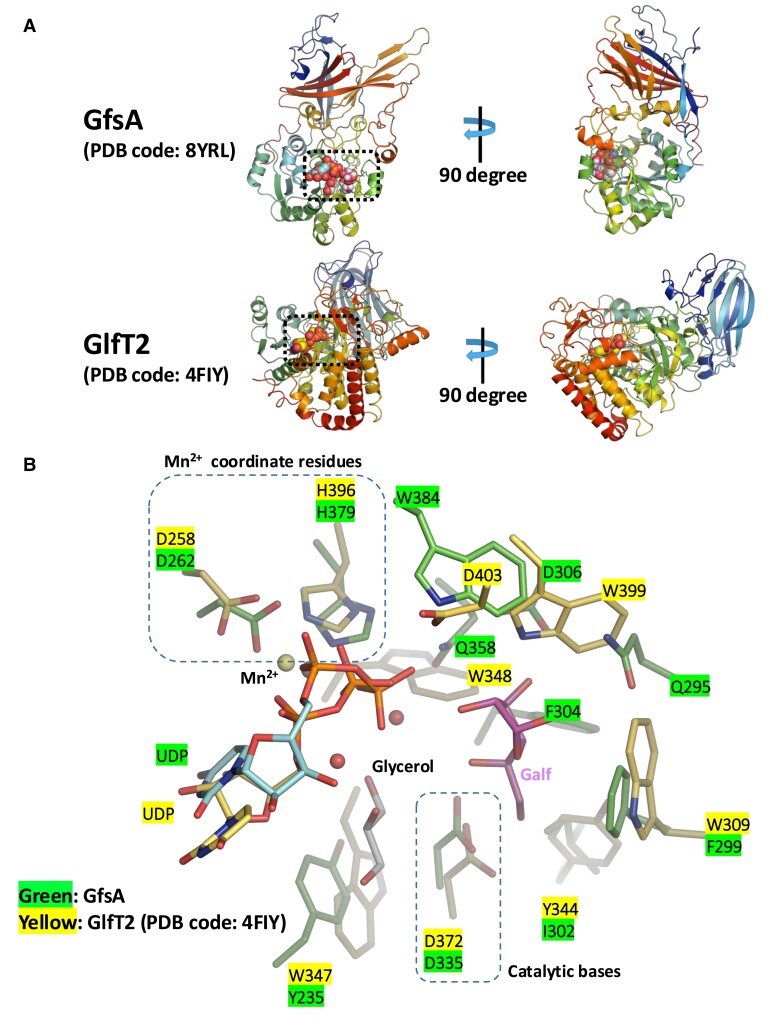
Comparison of the overall structures and active sites of GfsA and GlfT2. A) The overall structure of *A. fumigatus* GfsA (upper left, PDB code: 8YRL) and rotated 90° (upper right). The overall structure of *M. tuberculosis* GlfT2 (bottom left, PDB code: 4FIY) and rotated 90° (bottom right). B) Superimposed view of active sites of GfsA and GlfT2. Letters in yellow and green indicate the amino acid numbers derived from GfsA and GlfT2, respectively. The catalytic bases of GfsA and GlfT2 are D335 and D372, respectively. Mn^2+^ coordinate residues of GfsA are D262 and H379, and of GlfT2 are D258 and H396.

The catalytic mechanism of the galactofuranosyltransferase GlfT2, derived from *M. tuberculosis*, has already been reported (26, 27). Similar to GfsA, GlfT2, a member of the GT2 family of glycosyltransferases, uses UDP-Galf as the sugar donor and is responsible for the elongation of β-(1→5)-/β-(1→6)-galactofuranosyl chains. However, while GfsA is specific for β-(1→5) linkages, GlfT2 alternately catalyzes the formation of both β-(1→5) and β-(1→6) linkages. Furthermore, the overall structures of GfsA and GlfT2 are quite different (Fig. 6A): GfsA forms a dimer, while GlfT2 forms a tetramer (26). Any amino acid sequence homology is not present between GfsA and GlfT2. However, their active sites exhibit similar features, and superposition revealed the corresponding residues (Fig. 6B). The active base of GlfT2 is D372, and the DXD motif (D256, D258) along with H396 is involved in the orientation of Mn^2+^. In GfsA, the residue corresponding to D372 in GlfT2 is D335, which supports that the active residue in GfsA is D335. In GfsA, the DXD motif (D260 and D262) and H379 are involved in Mn^2+^ coordination. Although the primary structural sequence homology between GfsA and GlfT2 is low, the residues involved in Mn^2+^ recognition and general base catalysis are conserved, suggesting a similar enzymatic reaction mechanism.

### Distribution of GfsA to pathogenic fungi and conserved active sites

**Fig. 7.**
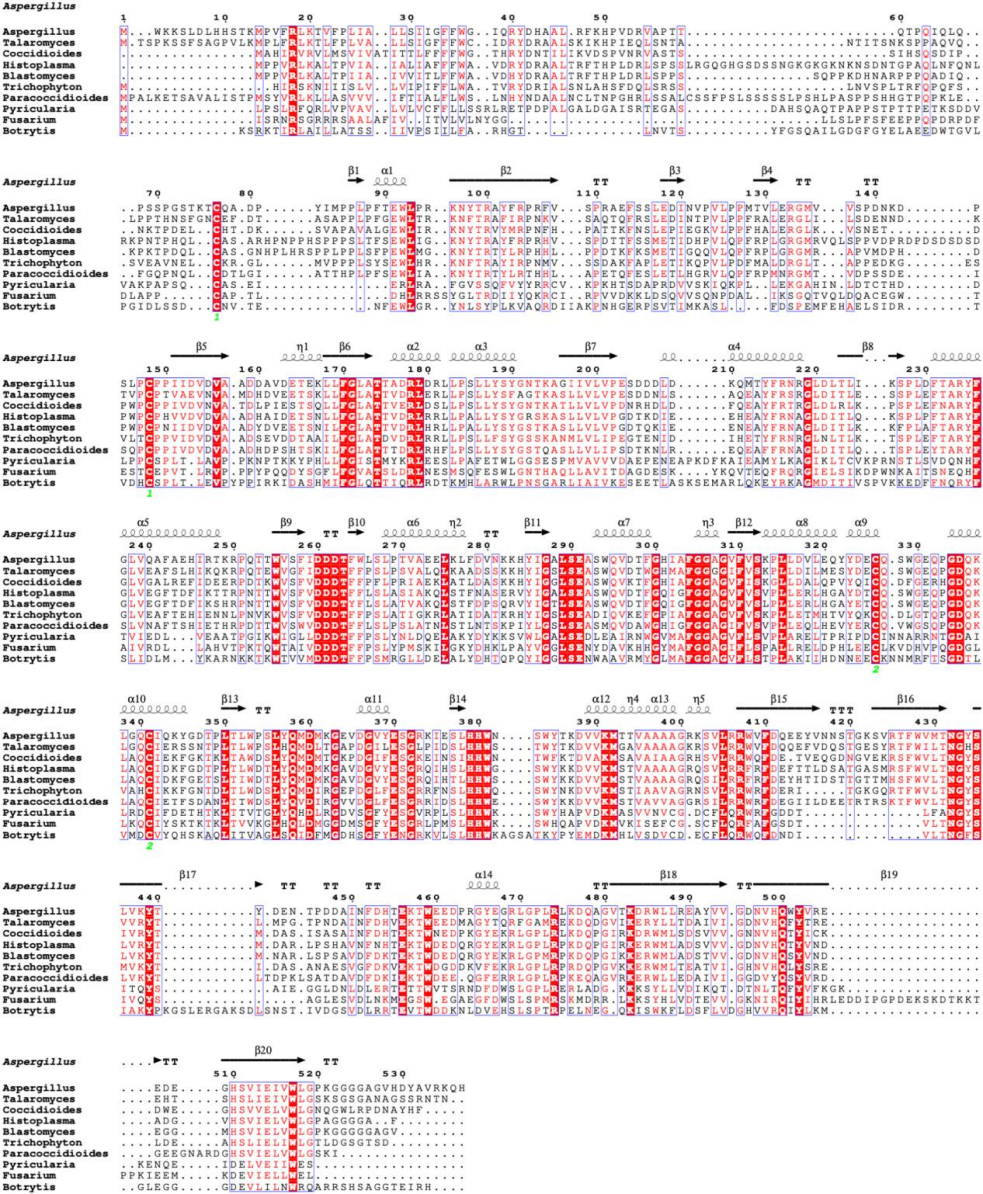
Structure-based sequence alignment of GfsA with known orthologous proteins. The sequence of GfsA is aligned to the orthologous proteins of pathogenic fungi. The secondary structure elements are drawn above the sequences. Filled triangles and circles under the sequences represent amino acids that coordinate to Mn^2+^ and form the active cavity, respectively. Green numbers indicate the Cys-pair residues forming three conserved disulfide bonds.

GfsA is an enzyme widely distributed in Ascomycota belonging to Pezizomycoina, which contains many notable pathogens. The GfsA of *Talaromyces marneffei*, *Coccidioides immitis*, *Histoplasma capsulatum*, *Blastomyces dermatitidis*, *Tricophyton rubrum*, *Paracoccidioides brasiliensis*, *Pyricularia oryzae*, *Fusarium oxysporum*, and *Botrytis cinerea* were selected from the genome database, and alignment analysis was performed (Fig. 7). Residues R178A, D260, D261, D262, F304, D335, D360, H379, and H380, which form the active cavity in the reaction, are conserved in all GfsA (Fig. 7), and the alanine substitutions of all these amino acids showed significantly reduced activity (Fig. 4A and B). To examine the conservation of residues in GfsA from a wide range of organisms, we conducted a computational analysis across GfsAs using the ConSurf server with standard parameters (Fig. S2B and C) and evaluating 68 unique GfsA protein sequences (28). We found that the residues forming the active cavity are conserved with a ConSurf score ≥8, indicating their high conservation in a much broader range of fungal species (Fig. S2B and C). Interestingly, highly conserved residues were found scattered throughout the binding surface of the dimer formed by GfsA, suggesting the importance of this for the GfsA function (Fig. S2B and C). The conservation of D260, D262, and H379 involved in Mn^2+^ coordination and the active base, D335, in all GfsAs indicates that those distributed in diverse species transfer Galf by the same mechanism, suggesting that a common compound could become an enzyme–activity inhibitor.

## Discussion

Filamentous fungi belonging to the subphylum Pezizomycotina can cause many infections in humans, livestock, and crops (1, 7). The recent emergence of fungal strains resistant to antifungal drugs necessitates the development of new antifungals with novel mechanisms of action. The main objective of this study was to contribute to drug development against filamentous fungal infections by elucidating the stereostructure and detailed catalytic mechanism of the glycosyltransferase enzyme GfsA through X-ray crystallographic analysis. GfsA was expressed and purified using *E. coli*, and crystal structures were determined for the Mn^2+^ complex and the Mn^2+^–UDP and Galf complex at a resolution of 2.37 Å (Figs. 1 and 2). Further structural features related to substrate recognition and catalytic activity were clarified through molecular modeling and mutant experiments (Figs. 3–5). Since the only reported structure of galactofuranosyltransferase is GlfT2 (26), GfsA–Mn^2+^–UDP and Galf complex structures provide a basis for understanding the galactofuranosylation.

The mechanism of action employed by most inverting enzymes involves an S(N)2-like single-displacement reaction, wherein the acceptor nucleophilic –OH attacks the anomeric carbon of the donor sugar, displacing the nucleotide moiety from the opposite face (30). GfsA specifically transfers β-Galf to the 5ʹ-OH of the acceptor substrate Galf through this catalytic mechanism (Fig. 5). In our study, the crystal structure revealed that the β-galactofuranoside had its 1ʹ-OH group oriented outward from the active site. This orientation allows the addition of continuous β-galactofuranosyl residues without steric hindrance, given the ample space for chain extension (Fig. S5A). There appears to be sufficient space to accommodate a β-galactofuranosylated mannan-core oligosaccharide with a single β-galactofuranosyl residue, which is another natural acceptor, in this position. In practice, the binding of a β-(1→5)-Galf acceptor with a chain length of three to the active site does not pose a steric hindrance, as ample space is available for further extension of the glycan chain (Fig. S5B). Thus, GfsA can catalytically extend Galf by up to seven sugar units (17), indicating a possible structure (Fig. S5B). GfsA specifically adds Galf and shows no catalytic activity with UDP-Glu or UDP-Galp substrates, indicating a strict recognition mechanism for Galf (16). However, although the obtained stereostructure revealed UDP binding, we still have not resolved the co-crystal structure with UDP-Galf. Consequently, we could not identify the amino acid residues that interact with the Galf part of UDP-Galf. Further research, including investigating the obtained co-crystal structure with UDP-Galf, is needed to elucidate the molecular mechanism of substrate specificity of donor substrates.

In GfsA, the active base D335 interacts with the 5ʹ-OH and 6ʹ-OH groups of the acceptor Galf. However, the proximity of the anomeric carbon of UDP-Galf to the Galf at position 5 enables this binding mode to be strictly regulated structurally, allowing a β-(1→5) linkage-specific galactofuranosylation reaction (Fig. 4). In GlfT2, interactions with the 5ʹ-OH and-6ʹ-OH groups of the acceptor Galf are mediated by the active base D372 and adjacent E300, Y344, and H396 (26, 27). For β-Galf-β-(1→6)-Galf-β-(1→5)-Galf as the acceptor substrate, the 5ʹ-OH interacts with E300, Y344, and H396, orienting this in the active site, with the 6ʹ-OH approaching D372 for Galf addition (27). With β-Galf-β-(1→5)-Galf-β-(1→6)-Galf, the 6ʹ-OH interacts with E300, Y344, and H396, orienting this in the active site, with the 5ʹ-OH approaching D372 for Galf addition (27). Distinct recognition mechanisms for acceptor Galf by GfsA and GlfT2 contribute to their different catalytic activities. GfsA strictly controls the binding of acceptor Galf through D335, whereas GlfT2 recognizes Galf more flexibly through multiple residues. GlfT2 is a glycosyltransferase belonging to GT2, while GfsA belongs to GT31. Since the primary structures and substrate recognition mechanisms of GfsA and GlfT2 are different, they probably did not acquire similar functions through horizontal transmission or divergent evolution. Although the overall structures of GfsA and GlfT2 are different, their active centers are similar, suggesting that they may have acquired similar functions through convergent evolution. This is analogous to the fact that hyaluronan synthases common to microorganisms and mammals each have the same functionality through convergent evolution (31). The official IUBMB enzyme classification for GlfT2 is EC 2.4.1.288, but since GlfT2 is responsible for a different enzymatic reaction than GfsA, a new IUBMB enzyme classification, EC 2.4.1.398, was assigned.

Similar to GT31 enzymes, GfsA transfers glycoside from a sugar nucleotide to an acceptor substrate in an inverting mechanism. GfsA contains a GT-A fold with the Mn^2+^ ion coordinated in the cavity. Currently, three 3D structures of enzymes belonging to three GT31s are registered in the Protein Data Bank (PDB). These enzymes are UDP-galactopyranose: glycoprotein-*N*-acetylgalactosamine β-1,3-galactosyltransferase (DmC1GalT1, 7Q4I) (32); UDP-*N*-acetylglucosamine: *N*-acetylgalactosaminide β-1,3-*N*-acetylglucosaminyltransferase (8TJC) (33); and UDP-*N*-acetylglucosamine: *O*-fucosylpeptide β-1,3-*N*-acetylglucosaminyltransferase (Manic Fringe; Mfng, 2J0A) (34). GfsA is the fourth example of a crystal structure of a GT31 family protein. These structures and the one of GfsA were superimposed so that their active center sites overlapped (Fig. S6). A common structural part of GT31 was evident in the overlap between residues 160 and 360 that form the GT-A fold (Fig. S6). The regions from the N terminus to residue 159 and from residue 361 to the C terminus formed a characteristic protein structure of GfsA. This unique domain may be involved in Golgi localization and interaction with other proteins. The GfsA-specific domain partly forms a dimer in contact with the GT-A fold domain of the paired GfsA. Since GfsA is involved in the biosynthesis of fungal-type galactomannan and O-mannose-type galactomannan along with mannosyltransferases, such as CmsA/Ktr4, CmsB/Ktr7, AnpA, and Mnt1 (35–38), a complex comprising these enzymes may be formed, which could affect glycan biosynthesis.

GfsA is a semiprocessive glycosyltransferase synthesizing Galf oligosaccharides with chain lengths ranging from 2 to 7 (17, 39). The active center sides of GfsA dimers face each other, which may help to synthesize relatively long Galf chains by increasing their affinity for the accepting substrate. Another known homomeric enzyme with mutually oriented active centers is ST8SiaIII, a polysialyltransferase, a feature that may be advantageous for synthesizing relatively long sugar chains (40). However, the active centers of the Mnn9 homodimers responsible for the biosynthesis of the fungal *N*-glycan outer chain structure face outward from each other and require further studies (41, 42).

Petit et al. (43) proposed conserved motifs I through V in GT31, partially consistent with our findings in the GfsA sequence using WebLogo 3 (Fig. S7). Motif I [(R/A/L)(R/A)xx(I/V/A)xx(T/S)W] was not conserved in the GfsAs, whereas motif II [(F/Y)(V/L/M)xxxDxD] contains a conserved metal-binding motif, DxD. Motif III [(L/Y/V)(Y/W/F)xG] contains a conserved G, as in the other GT31s, although this was positioned far from the active site. Motif IV [GxxYxxS] was largely conserved, with G306 located close to the active site, where it could interact with the donor substrate. Motif V [(E/D)DVxxGx(W/C)], which contains the catalytic base, contains a highly conserved D335 and C341. Although not included in motif V, G334 was highly conserved in GfsAs. In other GT31 motifs, G334 was replaced by either D or E. The carboxyl groups of these residues are known to interact with the sugar of the nucleotide; however, in GfsA, this interaction was lost upon replacing G. This loss of interaction may be a characteristic feature of galactofuranosyltransferases.

Structural comparisons between GfsA and other GT31 enzymes have been considered, particularly on their donor nucleotide sugar recognition mechanisms (Fig. S8). Our analysis revealed that GfsA most closely aligns with the GT31 enzyme DmC1GalT1 (PDB id: 7Q4I), with D335 in GfsA corresponding to D255 in DmC1GalT1, highlighting their functional similarity (Fig. S8). However, aside from the conserved glycine residue G305 in GfsA (G221 in DmC1GalT1), there is minimal conservation among the residues expected to recognize the donor sugar of the two enzymes. Unlike other GT31 enzymes that use conserved E or D residues to recognize pyranosides in motif V, GfsA family proteins conserve G334. Differences, such as the G334 residue (corresponding to E254 in DmC1GalT1) located before the catalytic D335, suggest that these variations might affect the recognition of five-membered (furanose) vs. six-membered (pyranose) sugar rings.

Loss of Galf-containing sugar chains can cause decreased mycelial growth rate, reduced conidia formation capacity, and decreased virulence (8–18). The degree of effect seems to vary from species to species, but inhibitors of GfsA could be used as pharmaceuticals or agrochemicals. Further exploration of inhibitors targeting GfsA would provide a foundation for developing new antifungal agents. Conversely, the mechanism by which GfsA selects native receptor substrates in the Golgi lumen and cooperates with other glycosyltransferases to biosynthesize glycans in vivo is still unknown and requires further studies.

## Materials and methods

### Strains and growth conditions

*Aspergillus* strains (Table S1) were used in this study and cultured on *Aspergillus* minimal medium (MM) (44).

### Protein expression and purification

As previously described, the expression plasmid encoding the GfsA protein (residues 43–537, minus the transmembrane domain sequence) with an N-terminal 6xHis-tag was constructed using pET15b-kai (16). The GfsA protein was expressed in Rosetta-gami B (DE3) (Agilent Technologies, Santa Clara, CA, USA) cells harboring the pET15b-GfsA. Starter cultures were inoculated into 700 mL of LB (Luria-Bertani) medium at 37°C with shaking at 180 rpm until the optical density at 600 nm reached 0.2, whereupon isopropyl β-D-1-thiogalactopyranoside was added to a final concentration of 1 mM. The culture was then grown at 18°C for 65 h while shaking at 180 rpm. Cells were centrifuged, and the pellets were resuspended in lysis buffer (50 mM Tris–HCl, pH 8.0, and 500 mM NaCl) and stored at −80°C. Cells were lysed via sonication and centrifuged to remove cell debris. The soluble fraction was added to a Ni-agarose column (Wako Fujifilm, Osaka, Japan), which was thoroughly washed with lysis buffer. The GfsA protein was eluted with lysis buffer containing 400 mM imidazole, further purified using a Superdex 200 16/60 pg column (Cytiva, Marlborough, MA, USA), equilibrated with 50 mM Tris–HCl (pH 8.0), and 500 mM NaCl, and brought to a concentration of 20 mg/mL.

### Generation of GfsA mutants

Single-point mutations were created in the putative GfsA catalytic domain *via* site-directed mutagenesis as follows: R178A, F231A, Y235A, D260A, D261A, D262A, D279A, Q295A, F299A, I302A, F304A, P333F, D335A, Q358A, D360A, H379A, H380A, and H384A. PCR was run with pET15b-GfsA as a template and the following primer pairs: GfsA-R178A-IF-F and GfsA-R178A-IF-R, GfsA-F231A-IF-F and GfsA-F231A-IF-R, GfsA-Y235A-IF-F and GfsA-Y235A-IF-R, GfsA-D260A-IF-F and GfsA-D260A-IF-R, GfsA-D261A-IF-F and GfsA-D261A-IF-R, GfsA-D262A-IF-F and GfsA-D262A-IF-R, GfsA-D279A-IF-F and GfsA-D279A-IF-R, GfsA-Q295A-IF-F and GfsA-Q295A-IF-R, GfsA-F299A-IF-F and GfsA-F299A-IF-R, GfsA-I302A-IF-F and GfsA-I302A-IF-R, GfsA-F304A-IF-F and GfsA-F304A-IF-R, GfsA-P333A-IF-F and GfsA-P333A-IF-R, GfsA-D335A-IF-F and GfsA-D335A-IF-R, GfsA-Q358A-IF-F and GfsA-Q358A-IF-R, GfsA-D360A-IF-F and GfsA-D360A-IF-R, GfsA-H379A-IF-F and GfsA-H379A-IF-R, GfsA-H380A-IF-F and GfsA-H380A-IF-R, and GfsA-W384A-IF-F and GfsA-W384A-IF-R. DNA fragments were amplified and circulized using a Fast Gene Gel/PCR Extraction Kit (NIPPON GENE, Japan) and an in-fusion HD cloning kit. The following plasmids were constructed as described above: pET15-GfsA(R178A), pET15-GfsA(F231A), pET15-GfsA(Y235A), pET15-GfsA(D260A), pET15-GfsA(D261A), pET15-GfsA(D262A), pET15-GfsA(D279A), pET15-GfsA(Q295A), pET15-GfsA(F299A), pET15-GfsA(I302A), pET15-GfsA(F304A), pET15-GfsA(P333F), pET15-GfsA(D335A), pET15-GfsA(Q358A), pET15-GfsA(D360A), pET15-GfsA(H379A), pET15-GfsA(H380A), and pET15-GfsA(H384A). All PCRs used KOD One (Toyobo Co. Ltd., Osaka, Japan), and an ABI PRISM 3100 Genetic Analyzer (Applied Biosystems, Foster, CA, USA) was used to confirm the DNA sequence analysis.

### Construction of GfsA expression vectors and single amino acid GfsA substitution mutants

The *GfsA* gene was cloned into pHSG396 and amplified with PCR using *A. fumigatus* A1151 genomic DNA as a template for the pHSG396-GfsA-F and pHSG396-GfsA-R primers (Table S2). Next, the amplified DNA fragment, digested by KpnI, was inserted into the KpnI site of pHSG396 to yield pHSG396-GfsA. The following single-point mutations were introduced into the GfsA catalytic domain *via* site-directed mutagenesis: D260A, D262A, D335A, D360A, H379A, and H380A. PCR was run using pHSG396-GfsA as a template with the following primer pairs: GfsA-D260A-IF-F and GfsA-D260A-IF-R, GfsA-D262A-IF-F and GfsA-D262A-IF-R, GfsA-D335A-IF-F and GfsA-D335A-IF-R, GfsA-D360A-IF-F and GfsA-D360A-IF-R, GfsA-H379A-IF-F and GfsA-H379A-IF-R, and GfsA-H380A-IF-F and GfsA-H380A-IF-R. Amplified DNA was purified and circulized with a Fast Gene Gel/PCR Extraction Kit (NIPPON GENE, Japan) and an in-fusion HD cloning kit. The following plasmids were constructed: pHSG396-GfsA, pHSG396-GfsA(D260A), pHSG396-GfsA(D262A), pHSG396-GfsA(D335A), pHSG396-GfsA(D360A), pHSG396-GfsA(H379A), and pHSG396-GfsA(H380A). The entire *gfsA* gene and single amino acid substitution mutants were PCR amplified using pHSG396-GfsA and its derivatives as a template for the pPTR-II-IF-GfsA-F and pPTR-II-IF-GfsA-R primers (Table S2). Amplified DNA was inserted into the SmaI site of pPTR-II (TAKARA, Japan), a filamentous fungal plasmid capable of autonomous replication, using an in-fusion HD cloning kit to yield pPTR-II-GfsA, pPTR-II-GfsA(D260A), pPTR-II-GfsA(D262A), pPTR-II-GfsA(D335A), pPTR-II-GfsA(D360A), pPTR-II-GfsA(H379A), and pPTR-II-GfsA(H380A).

### Galactofuranosyltransferase assay

The galactofuranosyltransferase activity was measured as previously described (17). p-Nitrophenyl β-D-galactofuranoside (pNP-Galf) was obtained from Toronto Research Chemicals (Toronto, Canada). Standard assays used 1.5 mM pNP-β-D-Galf acceptor substrate, 20 mM UDP-α-D-galactopyranose, purified Glf protein (UDP-α-D-galactopyranose mutase from *E. coli*, 10.6 ug), 20 mM NADH, and purified GfsA (3.0 µg) proteins in a 20-µL total volume. The mixtures were incubated at 30°C for 3 h, and the reaction was stopped by heating at 99°C for 5 min. Supernatants were analyzed via high-performance liquid chromatography with an amino column Shodex Asahipak NH2P-50 4E (250 × 4.6 mm, Showa Denko, Tokyo, Japan). p-Nitrophenyl derivatives were detected by their absorbance at 300 nm, and the number of transferred β-galactofuranose residues computed from the peak area was defined as the activity value.

### Crystallization

The hanging-drop vapor diffusion method was used to screen crystallization conditions with five kits (PEG/Ion, PEG/Ion-2, Crystal Screen, Crystal Screen 2, and Index) from Hampton Research. The protein concentration was ∼10 mg/mL. To prepare the iodide derivative, MnCl_2_ was added to final concentrations of 2 mM. Each drop comprised GfsA (0.5 µL) and screening reservoir solution (0.5 µL). After several rounds, 0.1 M Bis–Tris (pH 5.5) containing 28% (w/v) polyethylene glycol 3350 was identified as the optimal reservoir solution for GfsA crystallization. Crystals needed around 10–13 days to reach their full size. For the GfsA–UDP–Galf complex, UDP and MnCl_2_ were added to final concentrations of 2 mM. Crystals complexed with UDP and Mn^2+^ were soaked into the reservoir solution supplemented with 50 mM pNP-Galf.

### Phase and structure determination and refinement

The GfsA structure was determined with SAD using an iodide derivative. Mn^2+^-GfsA crystals were soaked for 30 s in a cryobuffer (20% glycerol) containing 1 M KI and then flash frozen with a cryosystem (Rigaku, Tokyo, Japan). A 51.1-fold redundant 2.92-Å dataset was collected at beamline BL45XU, SPring-8, Hyogo, Japan. The initial phase of the structure determination of the iodide derivative GfsA was performed using the SOLVE/RESOLVE programs in the PHENIX system (45, 46). Density modification was applied to the initial phase using RESOLVE (47); the partial model was built automatically using RESOLVE (48) and ARP/wARP (49) and modified manually using COOT (50). The initial model was revised several times by alternately adjusting the model and refinements using phenix_refine (51) and Refmac5 (52).

For the GfsA–UDP–Galf complex, crystals were cryoprotected in a reservoir solution supplemented with 20% (v/v) glycerol that was flash frozen. A 2.37-Å dataset was collected at beamline BL45XU, SPring-8, Hyogo, Japan. The complex structure was solved with molecular replacement using the initial model (53). Structures were revised using COOT (50), phenix_refine (51), and Refmac5 (52). Refinement statistics are summarized in Table 1. The quality of the structure was assessed using MolProbity (54). Molecular graphics were subsequently created using PyMOL. The pKa and protonation state at pH 7 for each amino acid in the GfsA structure were calculated using the H++ web server (http://biophysics.cs.vt.edu/H++) (55).

### Computational modeling of ES complexes

ES complexes were generated by manual modeling followed by energy minimization. For the UDP–Galf complex, the structure of the UDP–glycerol–Galf complex was first experimentally determined. Glycerol and surrounding water molecules were used to guide the manual positioning of the Galf moiety of UDP-Galf. During this process, the hydroxyl groups of the Galf part of UDP-Galf were aligned with the hydroxyl groups of glycerol and the positions of nearby water molecules in the UDP–glycerol–Galf complex. Additionally, the distance between the 5ʹ-hydroxyl group of the acceptor Galf and the C1 carbon of the Galf moiety in UDP-Galf was constrained to 2.5–3.0 Å to reflect the expected catalytic geometry.

The modeling was performed using the Molecular Operating Environment (MOE) software, and energy minimization was carried out using MOE's default force field, Amber10:EHT.

### Modeling of the trisaccharide complex

For the trisaccharide complex, two additional Galf residues were manually added to the UDP-Galf acceptor in the modeled ES complex, ensuring the formation of a β-(1→5) linkage, consistent with the enzymatic reaction. The energy minimization was also performed using MOE with the Amber10:EHT force field.

## Supplementary Material

pgae482_Supplementary_Data

## Data Availability

All data pertaining to this study have been presented in the manuscript. The atomic coordinates and structure factors of the crystal structure have been deposited in the PDB under accession code 8YRL.
