# Supplementary material for: Substrate binding and catalytic mechanism of UDP-α-D-galactofuranose: β-galactofuranoside β-(1→5)-galactofuranosyltransferase GfsA
Source: PNAS Nexus. 2024 Oct 25;3(11):pgae482. doi: 10.1093/pnasnexus/pgae482 (PMC11538602; doi:10.1093/pnasnexus/pgae482)
Supplement: pgae482_Supplementary_Data [file pgae482_supplementary_data.pdf]

PNAS nexus

Substrate binding and catalytic mechanism of UDP-α-D-galactofuranose: β-galactofuranoside β-(1→5)-galactofuranosyltransferase GfsA

Takuji Oka, Ayana Okuno, Daisuke Hira, Takamasa Teramoto, Yuria Chihara, Rio Hirata, Chihiro Kadooka, Yoshimitsu Kakuta

Takuji Oka and Yoshimitsu Kakuta
E-mails: oka@bio.sojo-u.ac.jp (T.O.) & kakuta@agr.kyushu-u.ac.jp (Y.K.)

This PDF file includes:

Supplementary text
Figures S1 to S8
Tables S1 to S2

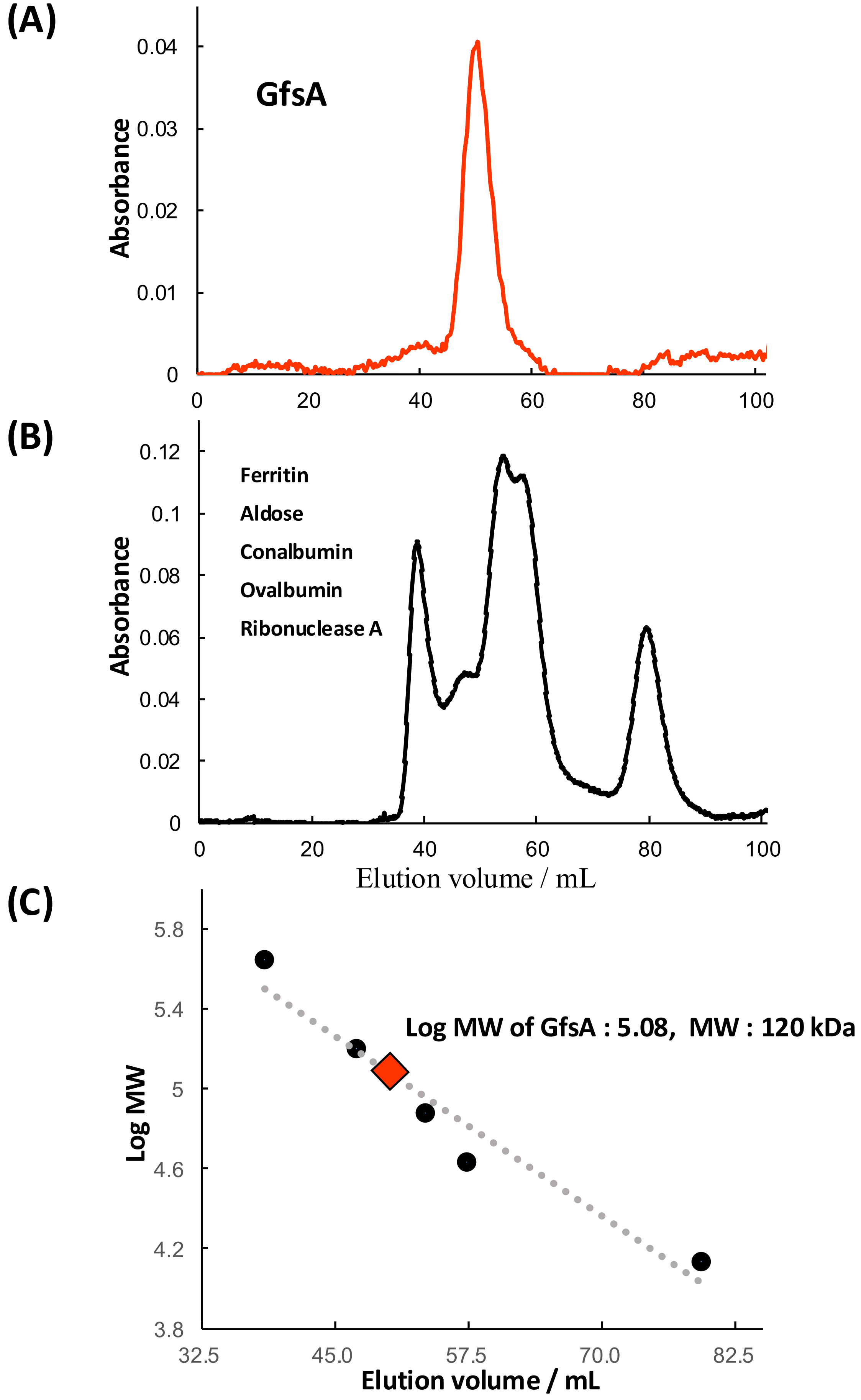

Fig. S1. Determination of the molecular weight of GfsA in solution by gel filtration chromatography. (A) Elution profile of GfsA by gel filtration chromatography (flow rate 1 mL/min) using HiPrep 16/60 Sephacryl S-200 HR. (B) Elution pattern of marker proteins, including ferritin, aldose, conalbumin, ovalbumin, and ribonuclease A, by gel filtration chromatography under similar conditions. (C) Calibration curve for the estimation of GfsA molecular weight.

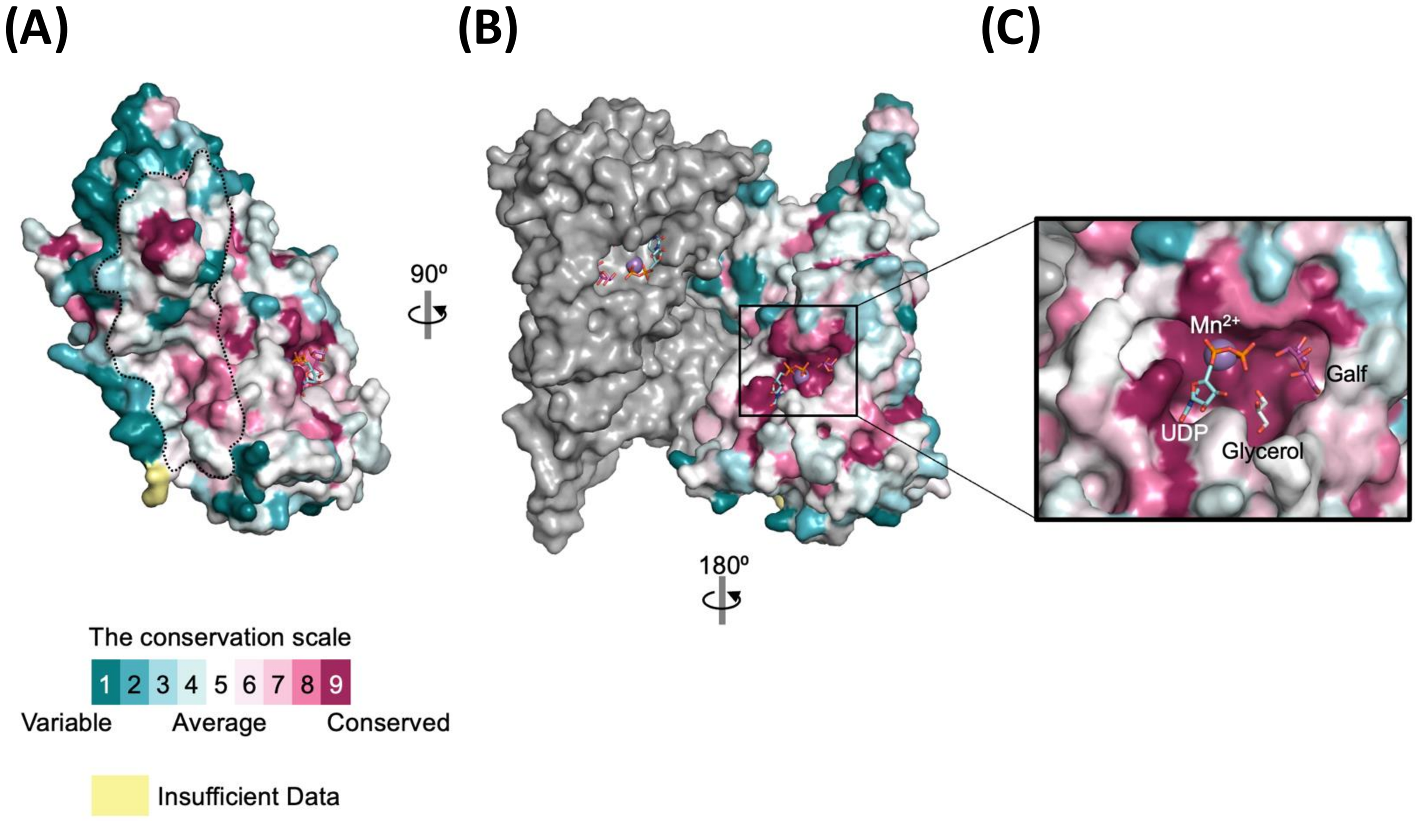

Fig. S2. Analysis of the regions conserved in GfsAs amino acid sequences from various fungi in the GfsA structure. Figures are drawn using GfsAs sequences from 68 fungi using Consurf server. The degree of amino acid conservatism is indicated by the Consurf score, which is numerically expressed from 1–8, and corresponds to the color from cyan to magenta in a direct proportion. (A) Consurf analysis of the GfsA monomer on the contact surface of the dimer. The dimer contact region is enclosed by dotted lines. (B) Consurf analysis of the GfsA dimer with active site in front. One monomer of the dimer is shown in grayscale. (C) Consurf analysis near the active site in GfsA.

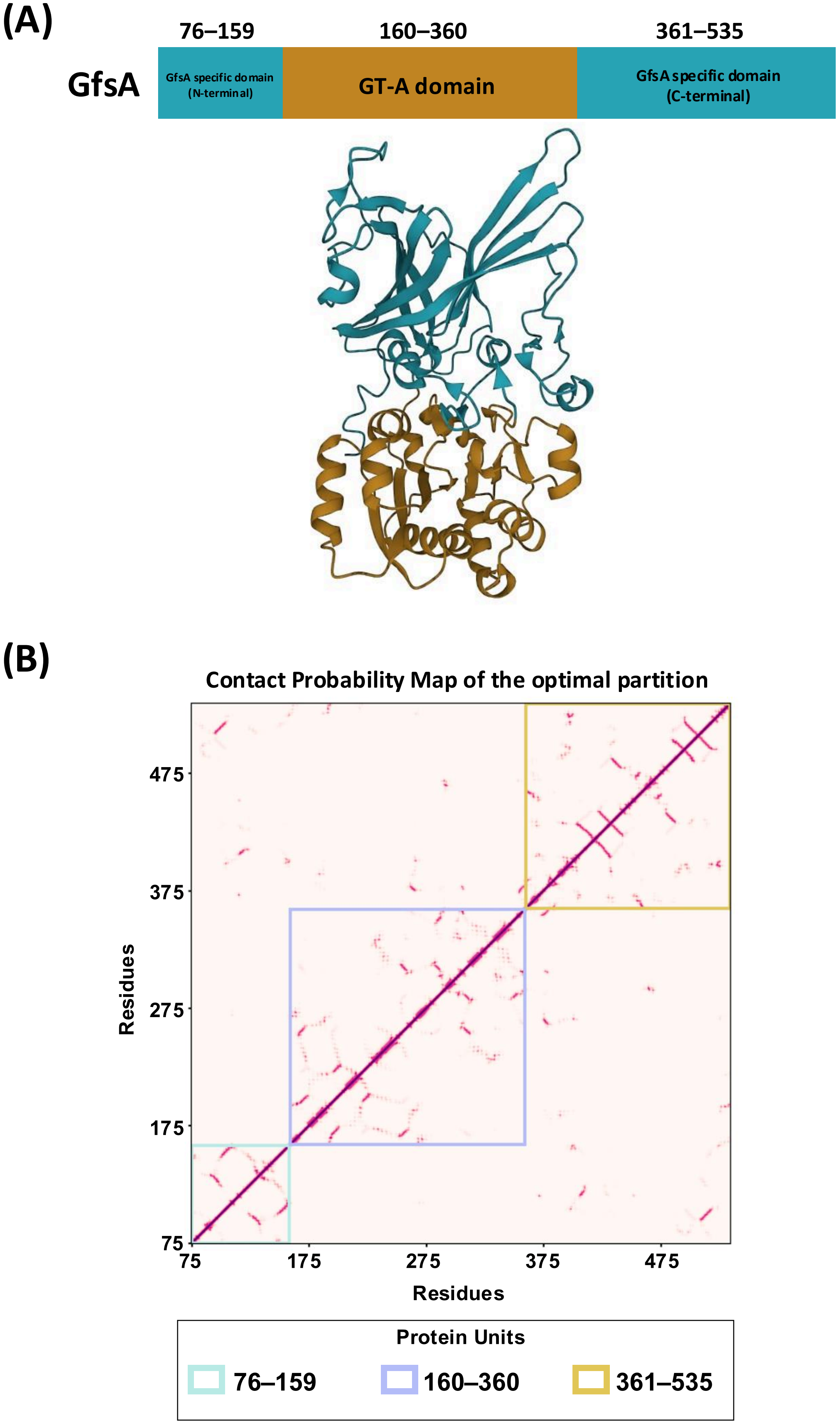

Fig. S3. Two domain structures of GfsA. (A) GT-A, also found in other GT31 family proteins, is in brown, and GfsA-specific domain, consisting of the N- and C-terminal regions, is in light blue. Domain assignment is based on the optimal partition using the SWORD2 partitioning algorithm. (B) Contact probability map of GfsA and the domain partition by SWORD2.

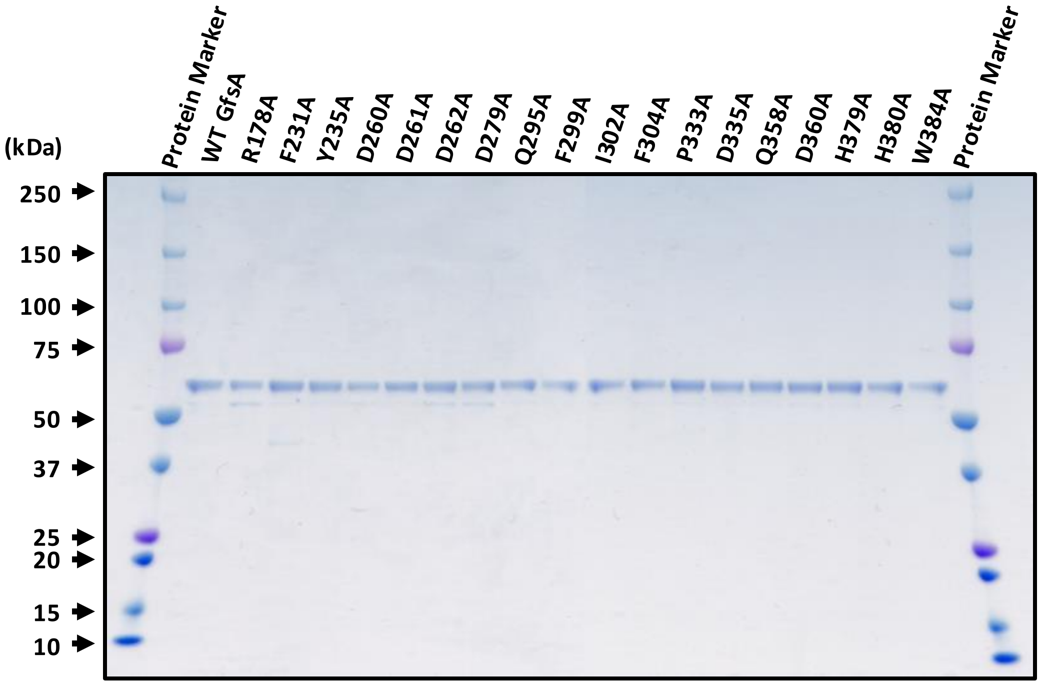

Fig. S4. SDS-PAGE of alanine substitutions of amino acids in purified *A. fumigatus* GfsA. Samples were loaded in the order of WT from left to right; GfsA, R178A, F231A, Y235A, D260A, D261A, D262A, D279A, Q295A, F299A, I302A, F304A, P333F, D335A, Q358A, D360A, H379A, H380A, and H384A. Each lane is loaded with 0.5 μg of protein. Proteins are stained with Coomassie Brilliant Blue.

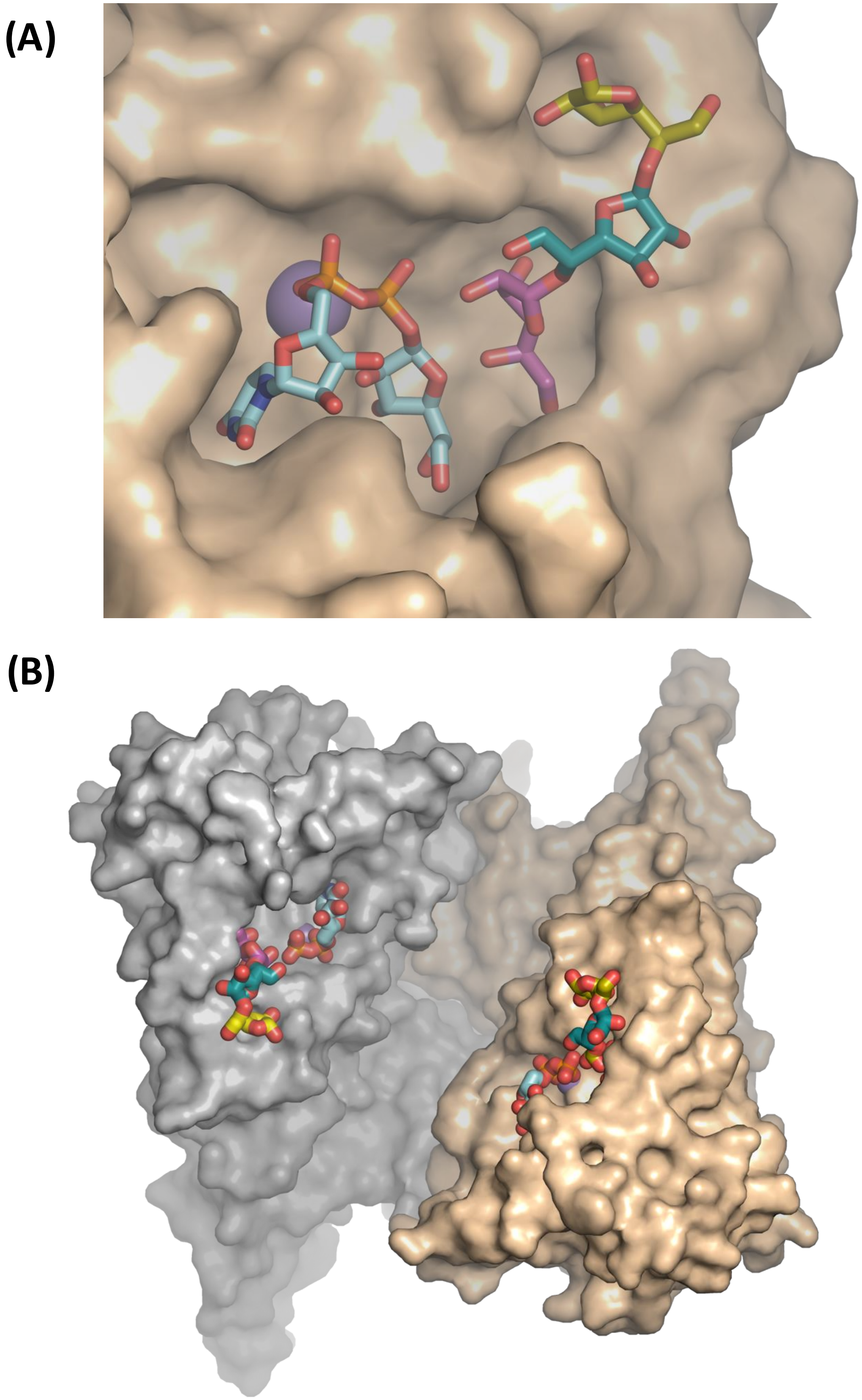

Fig. S5. Oligomeric sugar binding as a potential acceptor substrate to GfsA. By β-(1→5)-linking of Galf residues to the monosaccharide β-Galf position identified in the crystal structure, it is inferred that an acceptor substrate, e.g., β-Galf-β-(1→5)-Galf-β-(1→5)-Galf, could bind to GfsA. The carbon atoms of the two modeled Galf residues are shown in blue and yellow, respectively. Modeling of the acceptor substrate, β-(1→5)-Galf acceptor with a chain length of three, was performed using the Molecular Operating Environment (MOE) software. (A) Close-up view of the active center. (B) Overall structure of the dimer.

***Drosophila* C1GalT1**
(PDB code: 7Q4I)

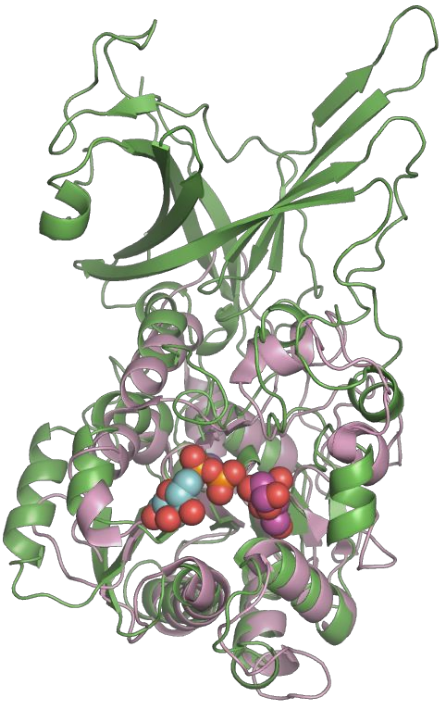

Mouse manic fringe
(PDB code: 2J0A)

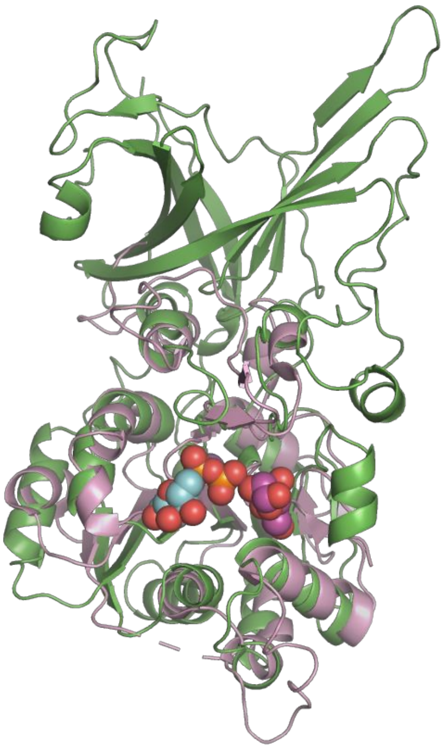

Human β1,3-*N*-acetylglucosaminyltransferase 2
(PDB code: 8TJC)

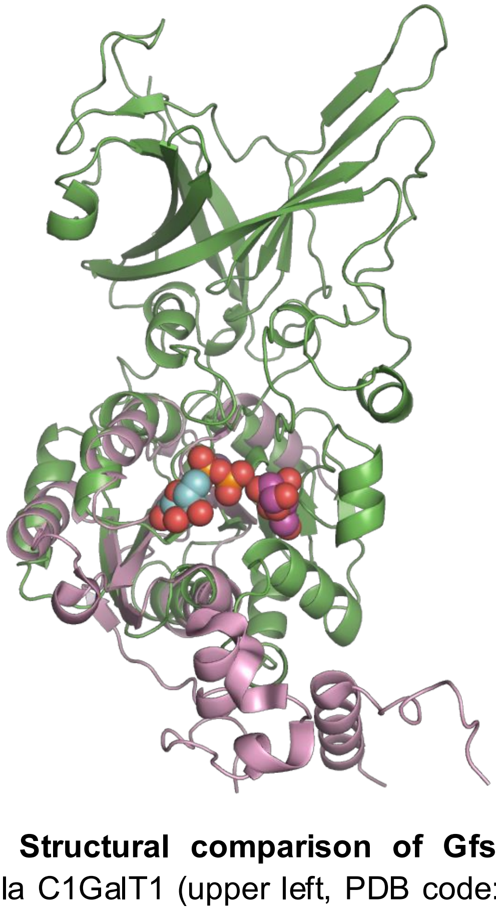

***Mycobacterium tuberculosis* GlfT2**
(PDB code: 4FIX)

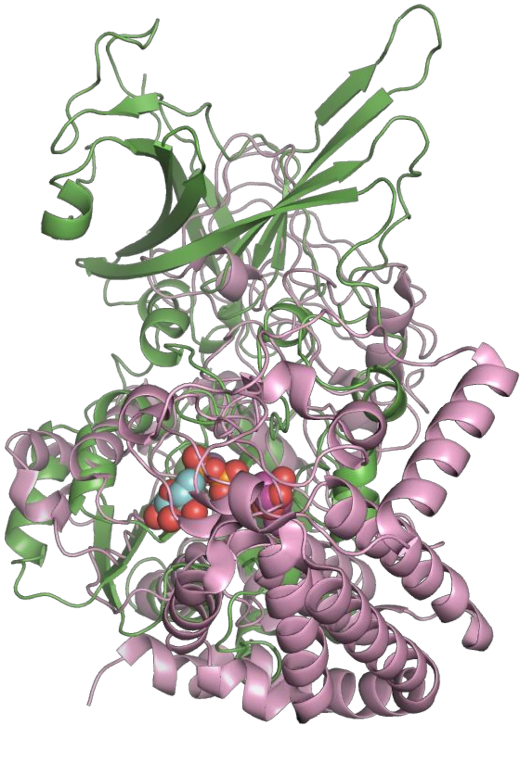

Fig. S6. Structural comparison of GfsA with other GT31 family proteins and GlfT2. Drosophila C1GalT1 (upper left, PDB code: 7Q4I), mouse manic fringe (upper right, PDB code: 2J0A), human β1,3-N-acetylglucosaminyltransferase 2 (lower-left PDB code: 8TJC), and *Mycobacterium tuberculosis* GlfT2 (bottom right, PDB code: 4FIX), respectively, are shown in pink ribbon models and superimposed with GfsA in green. GT31 family proteins and GlfT2 have some similarities with GT-A domain of GfsA but without a corresponding region to the GfsA-specific domain. PDB: Protein Data Bank

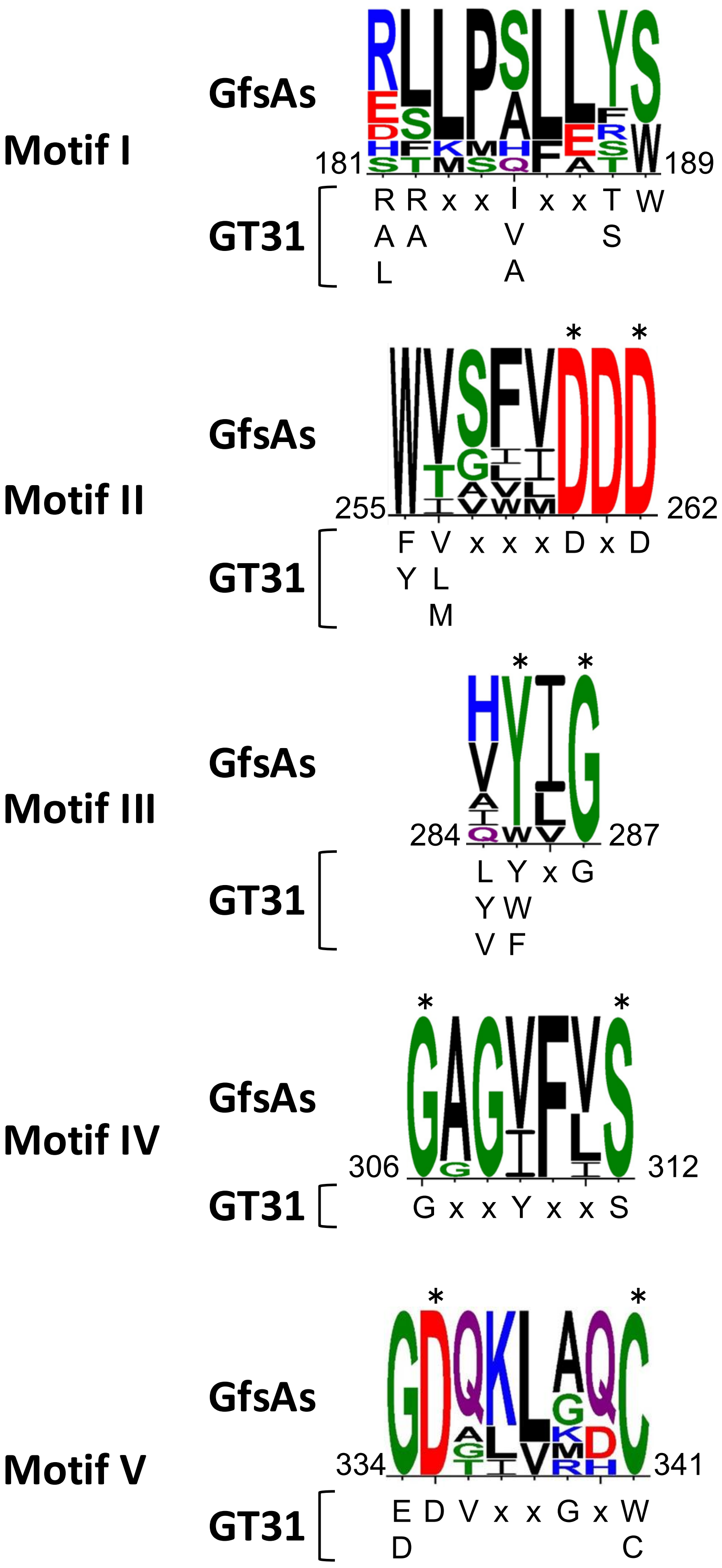

Fig. S7. Schematic representation of the five conserved motifs of the GfsA sequences among the animal β1,3-glycosyltransferases of the GT31 family. Graphical representation of the amino acid conservation at each position of the multiple alignments obtained using WebLogo 3 tool. Letter sizes are proportional to the degree of residue probabilities. The consensus sequences of the five motifs reported by Petit et al. are noted under each logo.

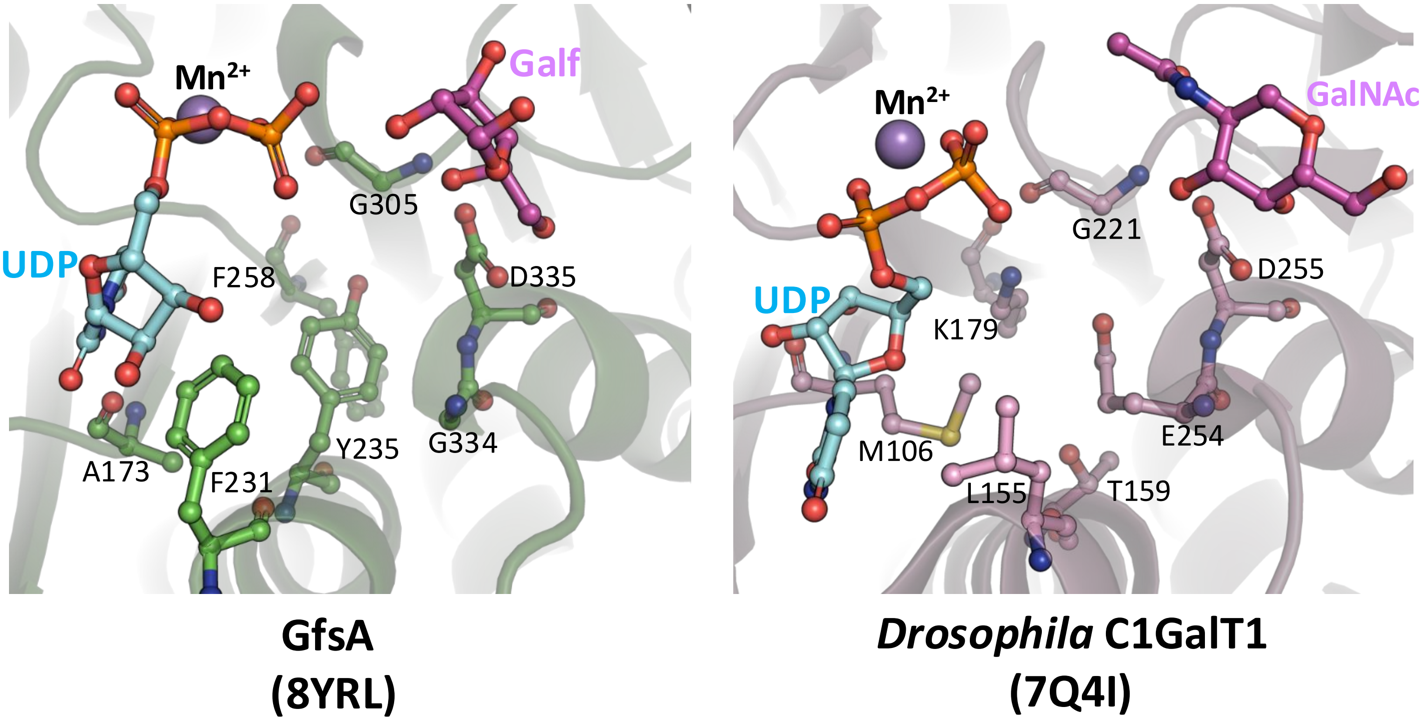

Fig. S8. Comparison between the active sites of GfsA and Drosophila C1GalT1. The catalytic sites of GfsA and Drosophila C1GalT1 (PDB code: 7Q4I) are superimposed. PDB: Protein Data Bank

Table S1. Strains used in the present study.

Strains	Genotype
Aspergillus fumigatus	
A1151	*pyrG* AF::Delta KU80
Aspergillus nidulans	
AKU89A	*biA1 argB2*::*argB akuB*::*AUR*$^{+}$
AKU89A+ pPTR-II	AKU89A harboring pPTR-II-AfGfsA
Δ*AngfsA*	*biA1 gfsA*::*argB akuB*::*AUR*$^{+}$
Δ*AngfsA* + pPTR-II	Δ*AngfsA* harboring pPTR-II-AfGfsA
Δ*AngfsA* + D260A	Δ*AngfsA* harboring pPTR-II-AfGfsA(D260A)
Δ*AngfsA* + D262A	Δ*AngfsA* harboring pPTR-II-AfGfsA(D262A)
Δ*AngfsA* + D335A	Δ*AngfsA* harboring pPTR-II-AfGfsA(D335A)
Δ*AngfsA* + D360A	Δ*AngfsA* harboring pPTR-II-AfGfsA(D360A)
Δ*AngfsA* + D379A	Δ*AngfsA* harboring pPTR-II-AfGfsA(D379A)
Δ*AngfsA* + D380A	Δ*AngfsA* harboring pPTR-II-AfGfsA(D380A)

Table S2. Oligonucleotide primers used in this study

pHSG396-GfsA-F	AAAAGGTACCTACGCCGCTTGCTACTTGGTTAG	This study
pHSG396-GfsA-R	AAAAGGTACCGGATTTTCCGTCCTGTGGCC	This study
pPTR-II-IF-GfsA-F	CTCTAGAGGATCCCCTACGCCGCTTGCTACTTGGTTAG	This study
pPTR-II-IF-GfsA-R	TCGAGCTCGGTACCCGGATTTTCCGTCCTGTGGCC	This study
GfsA-R178A-IF-F	GCTACCACTGCTGACGCACTGGATCGCCTTCTTCCT	This study
GfsA-R178A-IF-R	GTCAGCAGTGGTAGCAAG	This study
GfsA-F231A-IF-F	AAGTCTCCACTTGACGCAACCGCTCGTTATTTCGGC	This study
GfsA-F231A-IF-R	GTCAAGTGGAGACTTGATCAA	This study
GfsA-Y235A-IF-F	GACTTCACCGCTCGTGCATTCGGCCTGGTCCAGGCA	This study
GfsA-Y235A-IF-R	ACGAGCGGTGAAGTCAAG	This study
GfsA-D260A-IF-F	TGGGTCAGTTTTATCGCAGATGACACTTTCTGGTTATCCC	This study
GfsA-D260A-IF-R	GATAAAACTGACCCATGTGGT	This study
GfsA-D261A-IF-F	GTCAGTTTTATCGATGCAGACACTTTCTGGTTATCCCTT	This study
GfsA-D261A-IF-R	ATCGATAAAACTGACCCATGT	This study
GfsA-D262A-IF-F	AGTTTTATCGATGATGCAACTTTCTGGTTATCCCTTCCC	This study
GfsA-D262A-IF-R	ATCATCGATAAAACTGACCCAT	This study
GfsA-D279A-IF-F	GAACTGAAGCTGTTCGCAGTGAACAAAAAACATTATATTGGTGC	This study
GfsA-D279A-IF-R	GAACAGCTTCAGTTCCTCG	This study
GfsA-Q295A-IF-F	TCCGAAGCCAGCTGGGCAGTTGATACCTTCGGACACAT	This study
GfsA-Q295A-IF-R	CCAGCTGGCTTCGGACAA	This study
GfsA-F299A-IF-F	TGGCAGGTTGATACCGCAGGACACATTGCCTTCGGA	This study
GfsA-F299A-IF-R	GGTATCAACCTGCCAGCT	This study
GfsA-I302A-IF-F	GATACCTTCGGACACGCAGCCTTCGGAGGTGCTGGC	This study
GfsA-I302A-IF-R	GTGTCCGAAGGTATCAACC	This study
GfsA-F304A-IF-F	TTCGGACACATTGCCGCAGGAGGTGCTGGCGTATTT	This study
GfsA-F304A-IF-R	GGCAATGTGTCCGAAGGT	This study
GfsA-P333A-IF-F	TCATGGGGTGAGCAGGCAGGAGATCAGAAACTCGGCC	This study
GfsA-P333A-IF-R	CTGCTCACCCCATGACTG	This study
GfsA-D335A-IF-F	GGTGAGCAGCCCGGAGCACAGAAACTCGGCCAGTGC	This study
GfsA-D335A-IF-R	TCCGGGCTGCTCACCCCA	This study
GfsA-Q358A-IF-F	TGGCCCTCGCTCTACGCAATGGACATGAAGGGTGAAGT	This study
GfsA-Q358A-IF-R	GTAGAGCGAGGGCCAAAG	This study
GfsA-D360A-IF-F	TCGCTCTACCAGATGGCAATGAAGGGTGAAGTCGACG	This study
GfsA-D360A-IF-R	CATCTGGTAGAGCGAGGG	This study
GfsA-H379A-IF-F	AAGATTGAGTCTCTGGCACACTGGAACAGCTGGTATAC	This study
GfsA-H379A-IF-R	CAGAGACTCAATCTTCCGC	This study
GfsA-H380A-IF-F	ATTGAGTCTCTGCACGCATGGAACAGCTGGTATACCA	This study
GfsA-H380A-IF-R	GTGCAGAGACTCAATCTTCC	This study
GfsA-W384A-IF-F	CACCACTGGAACAGCGCATATACCAAGGATGTGGTAAAGATG	This study
GfsA-W384A-IF-R	GCTGTTCCAGTGGTGCAG	This study
